# 

**Published:** 1939

**Authors:** 


### CONTENTS.

| | Page |
|---|---|
| Some Recent Advances in Ophthalmology. By Anthony Palin, B.M., B.Ch., F.R.C.S. | 85 |
| Some Medical Considerations of Sports and Games. By J. E. Lovelock, B.M., B.Ch. | 103 |
| Spasmodic Rhinorrhœa. By E. Watson-Williams, M.C., Ch.M. | 109 |
| The Seborrhœic Dermatoses. By Norman Burgess, M.D., M.R.C.P. | 127 |
| Reviews of Books | 141 |
| Editorial Notes | 152 |
| Meetings of Societies | 156 |
| The Medical Library of the University of Bristol | 158 |
| Publications Received | 162 |
| Local Medical Notes | 163 |

